# Photon Pairs from Resonant Metasurfaces

**DOI:** 10.1021/acs.nanolett.1c01125

**Published:** 2021-05-10

**Authors:** Tomás Santiago-Cruz, Anna Fedotova, Vitaliy Sultanov, Maximilian A. Weissflog, Dennis Arslan, Mohammadreza Younesi, Thomas Pertsch, Isabelle Staude, Frank Setzpfandt, Maria Chekhova

**Affiliations:** † Max Planck Institute for the Science of Light, Staudtstraße 2, 91058 Erlangen, Germany; ‡ University of Erlangen-Nürnberg, Staudtstraße 7/B2, 91058 Erlangen, Germany; ¶ Max Planck School of Photonics, Albert-Einstein-Str. 6, 07745 Jena, Germany; § Institute of Applied Physics, Abbe Center of Photonics, Friedrich Schiller University Jena, 07745 Jena, Germany; || Fraunhofer Institute for Applied Optics and Precision Engineering, 07745 Jena, Germany; ⊥ Institute of Solid State Physics, Friedrich Schiller University Jena, 07743 Jena, Germany

**Keywords:** quantum optics, photon-pair generation, spontaneous parametric down-conversion, nonlinear metasurfaces, Mie-type resonances

## Abstract

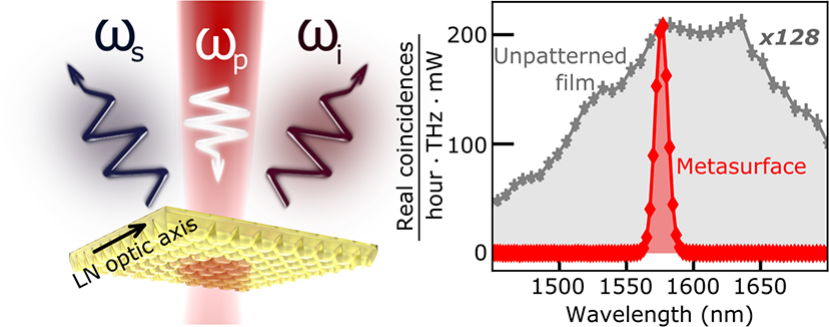

All-dielectric optical metasurfaces are a workhorse in nano-optics, because of both their ability to manipulate light in different degrees of freedom and their excellent performance at light frequency conversion. Here, we demonstrate first-time generation of photon pairs via spontaneous parametric-down conversion in lithium niobate quantum optical metasurfaces with electric and magnetic Mie-like resonances at various wavelengths. By engineering the quantum optical metasurface, we tailor the photon-pair spectrum in a controlled way. Within a narrow bandwidth around the resonance, the rate of pair production is enhanced up to 2 orders of magnitude, compared to an unpatterned film of the same thickness and material. These results enable flat-optics sources of entangled photons—a new promising platform for quantum optics experiments.

## Introduction

An important current tendency is the miniaturization of photonic devices toward multifunctional films with thicknesses in the nanometer range,^1^ so-called “metasurfaces”. Metasurfaces already found several applications in linear optics, such as ultrathin lenses,^2^ holograms,^3^ mode converters,^4^ and filter structures.^5^ Recently, they have also been established as a promising platform for nonlinear optics.^6^ The advantages of nonlinear flat-optics devices are their slim profile, ultrafast and broadband operation, and relaxed phase matching in frequency conversion.^7^ The latter leads to unprecedented freedom in the choice and engineering of nonlinear materials. Nanoscale sources with engineered nonlinearities and resonant field enhancement provide efficiencies of nonlinear frequency conversion as high as 1*%*^8^ and versatile nonlinear beam shaping.^9^ However, most of the previous work has been focused on the generation and control of classical light. The next natural step are quantum optical metasurfaces (QOMs) for the generation of quantum states of light through nonlinear processes at the nanoscale.^10,11^

While single-photon emitters have already been integrated into flat-optics platforms,^12^ the efficient generation of photon pairs within such structures remains a challenge. Pair generation has been reported using spontaneous four-wave mixing in carbon nanotube films,^13^ but the efficiency and signal-to-noise ratio were low. Another commonly used nonlinear process to create photon pairs is spontaneous parametric down-conversion (SPDC). In SPDC, a pump photon of higher frequency ω_p_ splits in two daughter photons, signal and idler, with lower frequencies ω_s_ and ω_i_, where energy conservation requires ω_s_ + ω_i_ = ω_p_. Recently, photon-pair generation by SPDC was demonstrated in ultrathin films of lithium niobate (LN) and gallium phosphide (GaP).^14,15^ However, despite the high bulk second-order susceptibilities χ^(2)^ of these materials, the achieved pair generation rates were modest: SPDC is based on the parametric amplification of the vacuum field,^16^ which is extremely weak. Furthermore, contrary to classical nonlinear processes like second-harmonic generation (SHG), the SPDC rate scales linearly with the pump power, and hence its efficiency cannot be enhanced by using a pulsed or focused pump.

To boost the efficiency of nanoscale SPDC, nanostructures with resonances at the signal and idler frequencies can be employed. Such structures feature an increased density of states, thus enhancing the vacuum field and enabling more efficient SPDC. This approach was already studied theoretically^17,18^ and experimentally^19^ for single nanoresonators exhibiting Mie-type resonances. Although the demonstrated results are promising, they are limited by the small total volume of the used resonators. Nonlinear metasurfaces—two-dimensional arrangements of such nonlinear nanoresonators—promise a better photon-pair generation rate.

Especially all-dielectric high-χ^(2)^ metasurfaces^20^ are a favorable platform for SPDC, because of their high damage threshold. Various resonance effects, such as Mie-type^21,22^ or Fano-type^23^ resonances and bound states in the continuum,^24^ have been shown to enhance SHG efficiency by several orders of magnitude.^23,25^ Because of the similarities between classical parametric frequency conversion and SPDC,^26−28^ comparable enhancements are expected for the latter.

**Figure 1:**
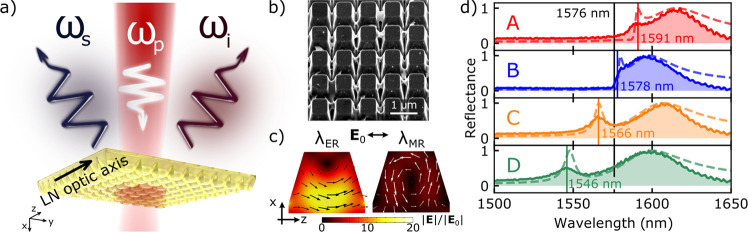
(a) Artist’s view of SPDC from a LN metasurface: the pump is incident from the substrate side, photon pairs are collected in reflection. Both the pump and the SPDC photons are polarized along the LN optic axis *z*. (b) Scanning electron microscopy (SEM) image of a fabricated metasurface, showing a periodic array of nanoresonators in the shape of truncated pyramids. (c) Electric field **E** distribution inside such a nanoresonator, as calculated in COMSOL Multiphysics at the electric resonances (λ_ER_) (left), and magnetic resonances (λ_MR_) (right). The incident field **E**_0_ is polarized along the LN optic axis, *z*-axis. Arrows show the electric field direction. (d) Experimental (solid lines, shading) and simulated (dashed lines) reflectance spectra of four QOMs with different resonance positions, further labeled as A, B, C, D; vertical black line marks the wavelength of degenerate SPDC. Vertical colored lines mark the positions of the electric resonances.

Here, we observe, for the first time, SPDC from resonant QOMs, schematically shown in Figure 1a. To emphasize the new possibilities of SPDC in metasurfaces, we generate photon pairs in reflection, which is difficult to achieve with many conventional SPDC sources. Because of the resonances, the photon pairs are emitted only into a narrow wavelength range, which opens a possibility to engineer their spectrum. Furthermore, within the emission bandwidth, we observe a two-orders-of-magnitude enhancement of the pair generation rate, compared to an unpatterned film of the same thickness as the metasurface, despite the fact that nanostructuring reduces the volume of the nonlinear material and our optics do not collect all photon pairs generated from the QOM.

## Results and Discussion

We generate photon pairs in QOMs made of LN, which is well known for its high second-order susceptibility χ^(2)^. Several recent works already demonstrated efficient SHG with LN nanoparticles^29^ and metasurfaces.^30−33^ LN is especially attractive for quantum nonlinear optics, because of its broad transparency range and relatively low fluorescence, compared to semiconductors like GaAs or GaP. In this work, we take advantage of its largest nonlinear tensor component χ_*zzz*_^(2)^.

Our metasurfaces are designed with fundamental magnetic and electric resonances for signal and idler photons. At resonance, the density of states is increased, enabling enhanced generation rates for photon pairs.^34^ Resonant enhancement at the pump wavelength could further increase the SPDC rate; however, since SPDC depends linearly on the pump power, the same effect can be achieved by modifying the pump properties.

We have fabricated QOMs on a 680-nm-thick x-cut LN-on-insulator film.^33^ The metasurfaces consist of nanoresonators in the shape of truncated pyramids with the side lengths at the half-height of ∼700 nm, arranged with a period of ∼900 nm as shown in the scanning electron microscopy (SEM) in Figure 1b. Our QOMs support two Mie-like resonances in the near-infrared wavelength range,^33^ further called “electric” and “magnetic” for the reasons discussed below. Their field distributions inside one nanoresonator are shown in Figure 1c, left and right panels, correspondingly. Using a custom-built white-light spectroscopy setup, we measured the reflectance of our QOMs for light polarized along the optic axis of LN (Figure 1d, solid lines). Each QOM exhibits a narrow electric resonance at shorter and a broad magnetic resonance at longer wavelengths, indicated by the maxima in the reflection spectra. The geometric parameters and the electric resonance wavelengths of all QOMs are listed in Table 1. With reducing the size/period of the pyramids, the resonances shift toward shorter wavelengths. Our experimental observations are corroborated by numerical simulations with the finite element method (dashed lines in Figure 1d). The short-wavelength electric resonance, although featuring several multipole components, is dominated by the electric dipole and quadrupole with the electric fields mainly in the plane of the metasurface (see the black arrows in Figure 1c). The long-wavelength magnetic resonance features electric field with a more complex structure and is dominated by the magnetic dipole. More details on the nanoresonator’s geometry and the resonances can be found in the Supporting Information (SI).

**Table 1:** Geometric Parameters and Resonant Wavelengths of QOMs

QOM	period (nm)	side length at half-height (nm)	electric resonance (nm)
A	930	700	1591
B	890	700	1578
C	930	690	1566
D	930	680	1546

Classical frequency-conversion experiments showed that electric-type resonances with fields in the metasurface plane can fully utilize the strongest component of the LN second-order susceptibility tensor χ_*zzz*_^(2)^, leading to improved conversion efficiencies.^33^ Furthermore, because the electric resonances in our metasurfaces have higher field enhancement than the magnetic resonances (see Figure 1c and the SI), they also provide a larger enhancement of the density of states. Therefore, for our SPDC experiments pumped at 788 nm, we chose QOMs with electric resonances near the degenerate photon-pair wavelength λ_deg_ = 2 × 788 nm = 1576 nm.

**Figure 2:**
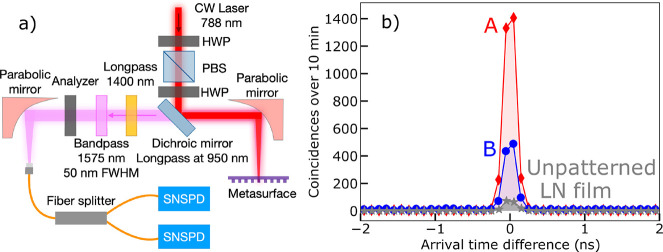
(a) Correlation experiment. A parabolic mirror focuses a cw pump into the QOM and collects backward-emitted SPDC. A dichroic mirror separates the SPDC radiation from the pump, and a 50 nm FWHM bandpass filter centered at 1575 nm transmits nearly degenerate photon pairs. Another parabolic mirror feeds the SPDC into a Hanbury Brown–Twiss setup formed by a fiber splitter and two superconducting nanowire single-photon detectors (SNSPD). (b) Coincidence histograms of degenerate SPDC from QOMs A and B, shown by red diamonds and blue circles, respectively. The lines are guides to the eye. Gray stars show the coincidence histogram from an unpatterned LN film of the same thickness as the nanoresonators. In all measurements, the pump power is ∼70 mW and the acquisition time is 10 min.

We pumped the QOMs from the substrate side with a continuous-wave (cw) laser at powers of several tens of milliwatts (Figure 2a). The pump laser was weakly focused using a parabolic mirror, resulting in a pump beam diameter of 6 μm on the QOM. The photon pairs emitted in the backward direction were collected using the same parabolic mirror. The filtering system comprised a long-pass filter for pump rejection, a bandpass filter of 50 nm full width at half-maximum (FWHM) bandwidth, centered at 1575 nm, and a polarization analyzer selecting polarization along the LN optic axis. Finally, the generated photons were coupled into a fiber and registered by two single-photon detectors in a Hanbury Brown-Twiss (HBT) setup (see Figure 2a and SI for more details). The collection numerical aperture (NA), determined by the fiber, was 0.14. Single-photon detection events from the two detectors were analyzed using a time-to-digital converter.

Figure 2b shows coincidence histograms, i.e., the numbers of two-photon detection events versus the difference in the photon arrival times, measured for ∼70 mW pump power over 10 min acquisition time, with the pump polarized along the LN optic axis. The red diamonds correspond to QOM A and the blue circles to QOM B. The peak in the middle indicates the simultaneous arrival of photons forming a pair. The coincidences-to-accidentals (peak-to-background) ratio (CAR) considerably exceeds 2 in both cases, which proves the generation of photon pairs in each QOM.^15^ The maximal obtained CAR is 361. The rates of real coincidences, found from the total number of coincidences after subtraction of the accidental coincidences, are 5.4 ± 0.1 Hz and 1.8 ± 0.1 Hz for QOMs A and B, respectively. The width of the coincidence histograms (180 ps) is determined by the timing jitter of the detectors.

We compared the photon-pair rates from the QOMs to that of an unpatterned LN film of the same thickness under the same experimental conditions (coincidence histogram shown by gray stars in Figure 2b). The peak values of the histograms measured in QOMs A and B are, respectively, 20 and 7 times higher than for the unpatterned LN. As shown below, we observe even a stronger enhancement of the pair generation rate by looking into a narrower spectral range.

**Figure 3:**
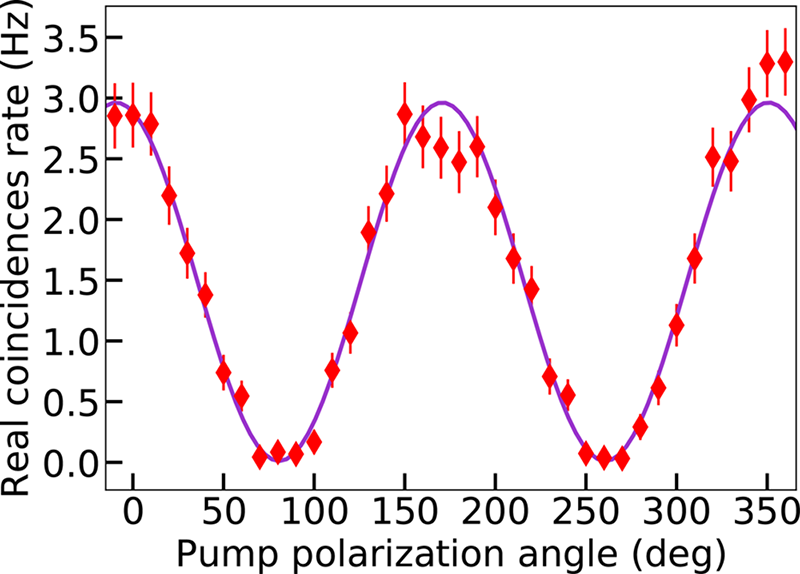
Real coincidence rate measured in QOM A versus the pump polarization angle, with respect to the LN optic axis. The pump power was ∼50 mW. The purple curve shows the theoretical cos^2^θ dependence.

To investigate the polarization dependence of SPDC, we have measured the coincidence rate while rotating the pump polarization angle and keeping the analyzer for signal and idler photons (Figure 2a) parallel to the LN optic axis. The result for QOM A, at the pump power ∼50 mW, is shown in Figure 3. The pair-generation rate scaled as cos^2^θ (purple curve), with θ being the pump polarization angle, with respect to the LN optic axis. For the analyzer oriented orthogonally to the LN optic axis, no photon pairs could be registered, regardless of the pump polarization. This behavior indicates that SPDC was indeed mediated by the χ_*zzz*_^(2)^ tensor component.

Generating entangled photons with tailored spectral correlations is one of the major tasks in quantum optics. To demonstrate the effect of the resonance on the observed enhancement of SPDC and its spectral distribution, we removed the bandpass filter and measured the spectrum of the backward-emitted photon pairs via single-photon spectroscopy.^35^ To this end, before the HBT setup, we inserted a single-mode fiber (Corning, Model SMF-28). Because of the group-velocity dispersion of the fiber, the signal and idler photons acquired a time delay that scaled as their wavelength separation 2π*c*(ω_s_^–1^–ω_i_^–1^):
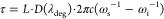
1

Here, *L* is the length of the fiber (*L* = 1 km) and *D*(λ_deg_) is its dispersion parameter at the degenerate wavelength (*D*(λ_deg_) ≈ 18.8 ps/(nm km)).^36,37^ Taking the energy conservation condition into account, eq 1 can map τ to the signal photon wavelength. By measuring τ with the time-to-digital converter, we obtained the SPDC spectrum^14,15^ with the resolution of 8.8 nm, determined by the timing jitter of the detectors.

**Figure 4:**
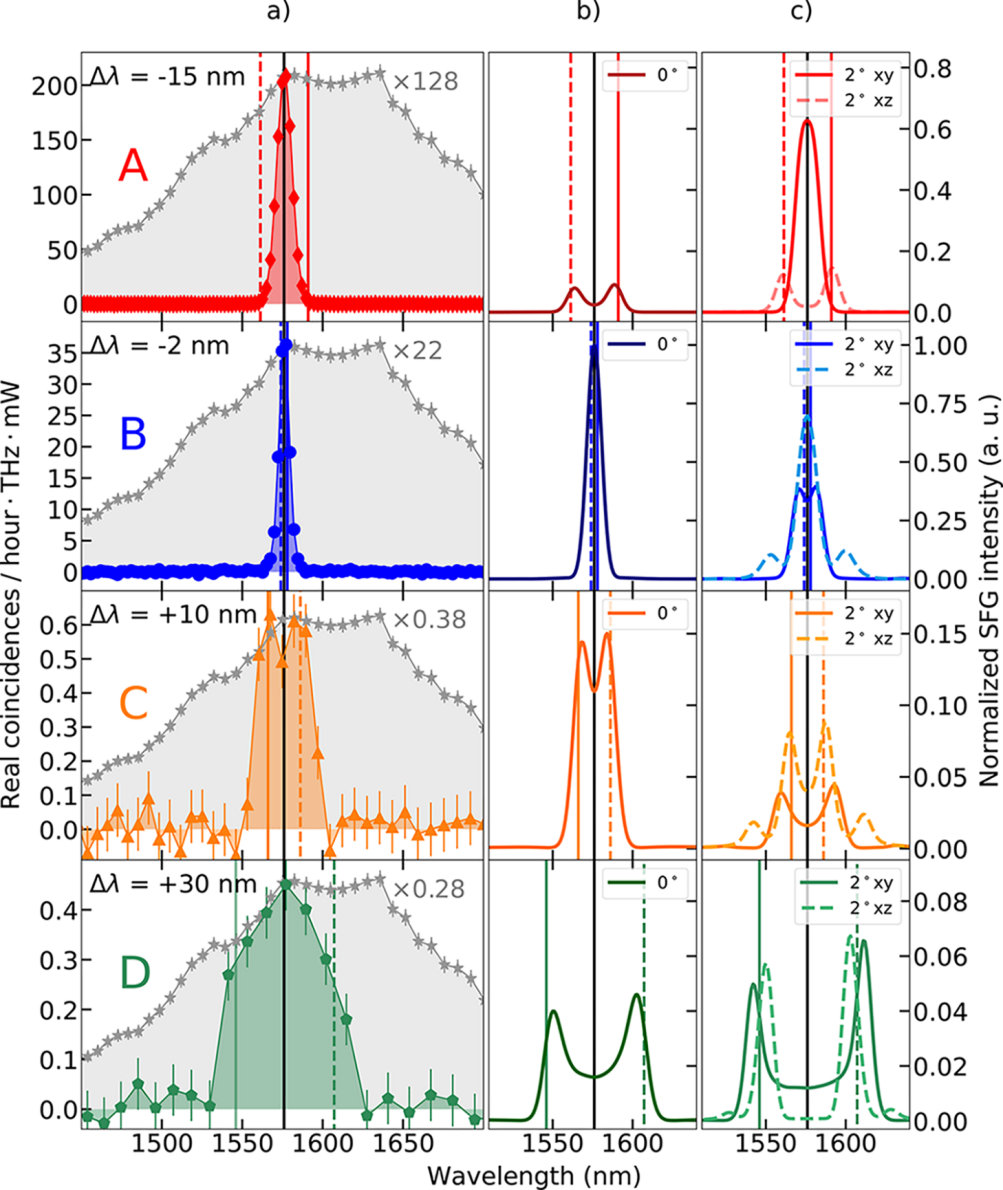
(a) Measured SPDC spectra from QOMs A (red), B (blue), C (orange), and D (green). Gray stars show the SPDC spectrum from the unpatterned LN film. (b) Spectra obtained through the numerical simulation of SFG at normal incidence. (c) SFG spectra calculated for the signal and idler incident at ±2° to the normal direction in the *xy* (solid curves) and *xz* (dashed curves) planes. Vertical colored lines mark the positions of the electric resonances, and the dashed vertical lines mark their conjugate wavelengths. The vertical black lines mark the wavelength of degenerate SPDC.

The measured SPDC spectra for QOMs A, B, C, and D are plotted in Figure 4a. All spectra are symmetric with respect to the wavelength of degenerate SPDC, λ_deg_ = 1576 nm, which is because our experiment cannot distinguish between the signal and idler photons. The measured SPDC spectra are all well-localized, in contrast to the spectrum of backward-emitted SPDC from the unpatterned LN film (gray stars in all panels). Here, we observed a much larger bandwidth, limited only by the spectral sensitivity of our setup. The spectrum of the LN film is broad, because the phase matching is relaxed^15^ and the vacuum field seeds SPDC uniformly over the spectrum.^16^

The width of the measured SPDC spectra is strongly dependent on the detuning between the degenerate wavelength λ_deg_ and the wavelength λ_ER_ of the electric resonance (Δλ ≡ λ_deg_ – λ_ER_). The latter is marked in each panel by a solid vertical line. Furthermore, the conjugate wavelength of the electric resonance, λ_conj_ = , is marked with a dashed vertical line. This is the wavelength where the partner photon to the photon emitted at resonance is detected. We see that each measured spectrum is bounded by the electric resonance wavelength and its conjugate, whereas no photon pairs are observed at wavelengths corresponding to the magnetic resonance. This is due to the larger field enhancement of the electric resonances, as well as the direction of their field, which is mainly along the LN optic axis and, thus, uses the χ_*zzz*_^(2)^ tensor component (see Figure 1c). The same effect was observed for classical frequency conversion in similar LN metasurfaces.^33^

The most striking feature of the measured spectra is the giant enhancement of SPDC rate within a narrow resonance bandwidth. For QOM A, degenerate photon pairs are emitted at a rate 130 times higher than that for the unpatterned LN film. The moderate advantage (20-times higher) observed in Figure 2b resulted from integrating the spectrum over the 50 nm bandwidth of the filter, while the resonance is not broader than 10 nm.

The strong enhancement is expected for QOMs with small detuning of the electric resonance from degeneracy, because efficient pair generation requires a high density of states at both signal and idler wavelengths.^34^ This is indeed true for QOM B, but we see an additional feature: the enhancement is stronger for metasurfaces with resonances red-detuned from degeneracy. For instance, although the resonance of QOM A (Δλ = −15 nm) is much more detuned from degeneracy than that of QOM B (Δλ = −2 nm), it is more efficient, whereas QOM C (Δλ = 10 nm) has a smaller detuning, but is very inefficient. This behavior can be understood by recalling that, in an ultrathin source, SPDC photons are emitted within a broad angle.^38^ However, for photons propagating at nonzero angles to the metasurface, the electric resonance gets blue-shifted, as we have shown by linear transmission measurements and simulations (see the SI). For our collection NA of 0.14, the resonances corresponding to the signal and idler waves with the largest angles can be blue-shifted by up to 80 nm. The total SPDC signal then is an average over SPDC spectra emitted in different directions, thus corresponding to different resonance positions. Effectively, the resonances contributing to the measured SPDC spectra are blue-shifted. As a result, collection of SPDC from a broad angle should give “preference” to QOMs with resonances red-detuned with respect to the degenerate wavelength.

To corroborate the experiments, we performed numerical simulations of sum-frequency generation (SFG) for signal and idler plane waves normally incident on the sample from the substrate side. According to the quantum-classical correspondence,^19,27,28^ the SFG efficiency is proportional to the rate of SPDC into the same modes. To obtain a full SFG spectrum, this calculation was performed for several signal and idler wavelengths satisfying energy conservation (see the SI for more details). In Figure 4b, we plot the normalized intensity of SFG emitted in the backward direction (reversed geometry to the one shown in Figure 1a for SPDC) for signal and idler with normal incidence. These simulations confirm the absence of the magnetic resonance contribution and the dependence of the spectral width on the resonance detuning.

Accounting for the whole collected solid angle of SPDC emission is beyond our computation capabilities; however, we performed SFG simulations for signal and idler propagating at ±2° to the normal in the *xy*- and *xz*-planes (Figure 4c). We see that, for QOM A, this small tilt in the *xy*-plane leads to a strong increase in the SFG efficiency and a spectral narrowing, since the electric resonance is shifted to the degenerate wavelength. Since the SPDC emission from QOM A will be dominated by these high-efficiency angles, this can explain the observed resonant narrowband enhancement. To explain why QOM A is more efficient than QOM B, we should keep in mind that large resonant enhancement for QOM A can occur not only for directions ±2° in the *xy*-plane, but for a large part of a ring in the collection aperture. In contrast, for QOM B, the resonant enhancement of SPDC happens just for a single direction at angle 0°. At any other direction, the SPDC efficiency is reduced. Hence, a smaller number of signal and idler modes benefits from the enhancement, which can explain why QOM B is less efficient than QOM A. Other properties of the fabricated sample, such as varying resonance strengths and different properties at the pump wavelength, can also contribute to the observed different efficiencies.

Meanwhile, QOMs C and D show a broader integrated spectrum, as the spectra at higher angles are also broadened. For QOM D, the simulations can qualitatively explain the measured SPDC bandwidth, but not the spectral distribution. We attribute this to measurement instabilities: this is the QOM with the weakest SPDC signal, and the measurement was performed over 5 days when the sample might have been slightly displaced and ambient conditions might have changed.

To conclude, we observed photon pairs generated via SPDC in resonant metasurfaces. Importantly, our experiment was the first-time observation of pronounced two-photon coincidences (CAR > 2) for SPDC in the reflection geometry. The photon-pair generation was strongly enhanced by the electric resonance: within its narrow bandwidth, enhancement by a factor of 130 was measured. Furthermore, the spectral width of the emitted photon pairs could be controlled through the detuning between the electric resonance and the SPDC degenerate wavelength, although the generation efficiency decreases with increasing detuning. Interestingly, the best performance was observed for a metasurface with the resonance red-shifted from the degenerate wavelength. This was due to the collection of photon pairs emitted at nonzero angles, for which the electric resonances shifted toward shorter wavelengths.

These results are very different from the ones we obtained earlier in the “transmission” geometry, where SPDC spectra showed dips instead of peaks at the resonance wavelengths and no enhancement.^39^

The actual enhancement of SPDC in resonant QOMs is even higher if we take into account the pump diffraction. Since the QOM period is larger than the pump wavelength, diffraction into the first orders occurs, the zeroth order carrying only a fraction of the incoming pump power.^33^ Photon pairs can be generated by each diffraction order of the pump; however, with our NA, we only collect pairs corresponding to the zeroth order. We believe that the measured photon-pair rate could be considerably increased by using optics with higher NA or modifying the QOM design. Furthermore, the resonant modes of the QOM could be optimized to emit photon pairs exclusively in the forward or backward direction, thus enabling more efficient SPDC in either reflection or transmission geometries.^18^

Our results are a first step toward the use of nonlinear metasurfaces as versatile sources of photon pairs. Apart from the spectral control that we demonstrated, QOMs will also enable far-reaching control of the spatial properties of SPDC, leading to unprecedented possibilities for the creation of complex two-photon quantum states.

## Supporting Information Available

Details on the linear simulations, linear spectra of selected metasurfaces in different geometries (reflection and transmission) for normal and oblique incidence, the multipole decompositions, the details on the field enhancement, description of the correlation experiment in reflection, and the details on simulations of sum-frequency generation (PDF)
