# Supplementary material for: Photon Pairs from Resonant Metasurfaces
Source: Nano Lett. 2021 May 10;21(10):4423–9. doi: 10.1021/acs.nanolett.1c01125 (PMC8289292; doi:10.1021/acs.nanolett.1c01125)
Supplement: Supplementary file 1 — nl1c01125_si_001.pdf [file nl1c01125_si_001.pdf]

Photon Pairs from Resonant Metasurfaces: Supporting Information

Tomás Santiago-Cruz,[*,†,‡,¶,#] Anna Fedotova,[§,#] Vitaliy Sultanov,[†,‡] Maximilian A. Weissflog,[§,¶] Dennis Arslan,[§] Mohammadreza Younesi,[§] Thomas Pertsch,[§,¶,‖] Isabelle Staude,[§,⊥] Frank Setzpfandt,[§] and Maria V. Chekhova[†,‡,¶]

†*Max Planck Institute for the Science of Light, Staudtstraße 2, 91058 Erlangen, Germany.*

‡*University of Erlangen-Nürnberg, Staudtstraße 7/B2, 91058 Erlangen, Germany.*

¶*Max Planck School of Photonics, Albert-Einstein-Str. 6, 07745 Jena, Germany.*

§*Institute of Applied Physics, Abbe Center of Photonics, Friedrich Schiller University Jena, 07745 Jena, Germany.*

‖*Fraunhofer Institute for Applied Optics and Precision Engineering, 07745 Jena, Germany.*

⊥*Institute of Solid State Physics, Friedrich Schiller University Jena, 07743 Jena, Germany.*

#*These authors contributed equally.*

E-mail: jose-tomas.santiago@mpl.mpg.de

Contents

1 Linear simulations **3**

2 Linear spectra **4**

2.1 Normal incidence 4

2.2 Oblique incidence 5

3 Multipole decomposition **8**

4 Field enhancement **9**

5 Correlation experiment in reflection **10**

6 Simulations of sum-frequency generation **11**

1 Linear simulations

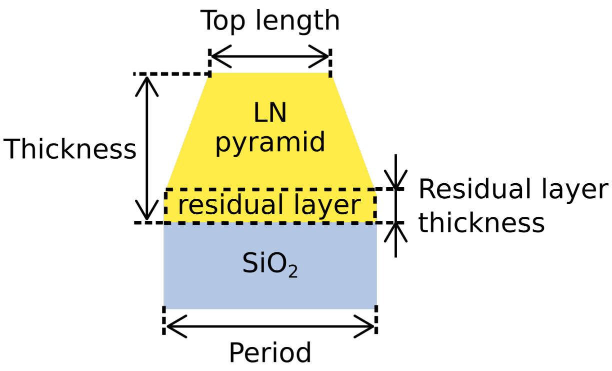

Figure S1: Sketch of one unit cell used in simulations.

To support our experimental results we performed numerical simulations using the finite-element method software COMSOL Multiphysics. In our metasurfaces the nanoresonators were designed as nanocubes with side lengths/periods: QOM A 700/930 nm, QOM B 700/890 nm, QOM C 690/930 nm, QOM D 680/930 nm. But fabrication resulted in truncated pyramids on residual lithium niobate (LN) layer (see Fig. 1(b)). We modelled our metasurfaces as a periodic unit cell containing a LN pyramid and LN residual layer on the SiO_2 substrate as shown in Fig. S1. In Table 1 we provide the four model parameters used in simulations. *Period* is the period of the unit cell, *Thickness* is the thickness of the LN nanoresonator formed by LN pyramid and LN residual layer, *Top length* is the pyramid's top length and it's base length was equal to the period value, *Residual layer thickness* is the thickness of the LN residual layer under the pyramid.

Table 1: Geometric parameters used in simulations.

Metasurface	Period, nm	Thickness, nm	Top length, nm	Residual layer thickness, nm
QOM A	930	680	556	110
QOM B	900	680	594	140
QOM C	930	680	551	75
QOM D	930	680	539	54

2 Linear spectra

2.1 Normal incidence

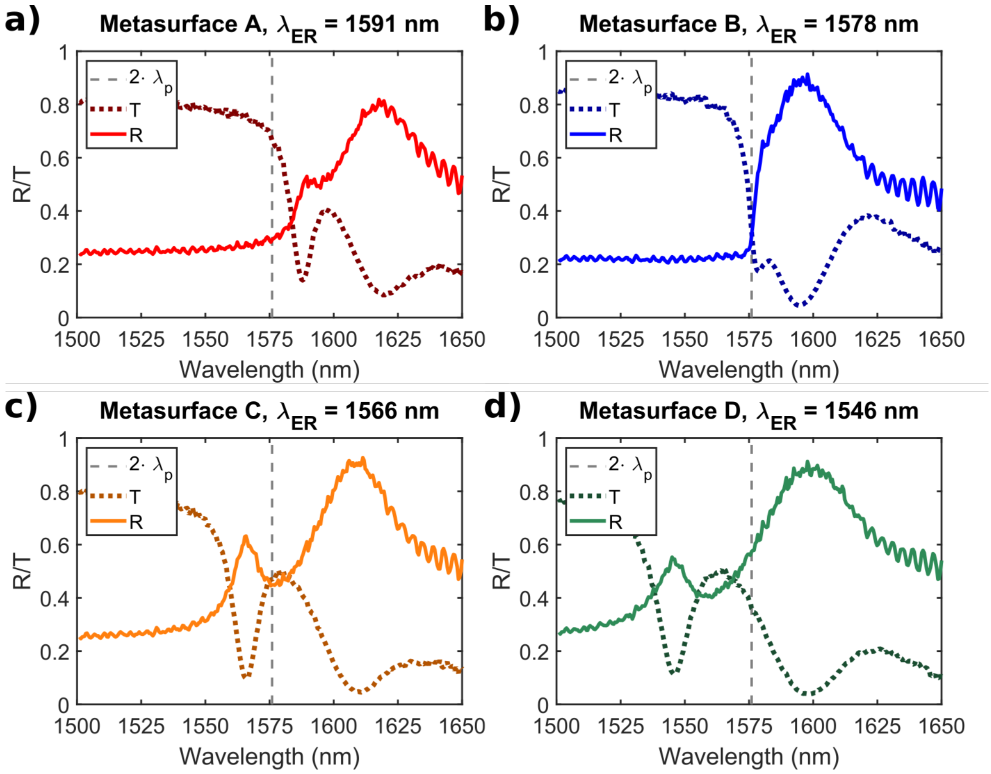

Figure S2: Experimental reflectance (solid lines) and transmittance (dotted lines) spectra for QOMs of interest, spectral positions of electric resonances λ_{ER} are noted in the title of each QOM. The polarization is along the LN optic axis. The dashed gray line indicates the degenerate photon-pair wavelength.

We measured the reflectance and transmittance spectra of our quantum optical metasurfaces (QOMs) using a custom-built white-light spectroscopy setup with collimated and linearly polarized excitation. Two Mie-type resonances appear in transmittance spectra (dotted lines) as dips and in reflectance spectra (solid lines) as peaks as can be seen in Fig. S2. Their spectral positions are summarized for all QOMs in Table 2. Since the resonances at shorter wavelengths (λ_{ER}) are dominated by electric multipole components and the resonances at longer wavelengths by magnetic dipole contribution (as discussed below), we refer to them simply as *electric resonances* and *magnetic resonances*. In reflection, the electric

resonances appear to be broader and flatter than in transmittance, which can be attributed to the scattering losses due to fabrication imperfections. Small fringes in the spectra are caused by Fabry-Pérot-like interferences in the setup.

As the linear spectra showed some constant background coming from scattering, in Fig. 1(c) it was removed by subtracting from the QOM reflectance R_{QOM} the reflectance of the wafer R_{waf} and normalizing the result to the maximum: $(R_{\mathrm{QOM}} - R_{\mathrm{waf}})/\max(R_{\mathrm{QOM}} - R_{\mathrm{waf}})$.

Table 2: Positions of Mie-type resonances.

Metasurface	Electric resonance, nm	Magnetic resonance, nm
QOM A	1591	1617
QOM B	1578	1595
QOM C	1566	1609
QOM D	1546	1597

2.2 Oblique incidence

In thin films photon pairs created via SPDC are generated within a broad angle.[1] In general, this also applies to our experiment but our optical system collects only pairs within the NA=0.14. However, for photons generated under an angle the resonances are effectively shifted. In Fig. S3 we plot experimental and simulated linear transmittance spectra for one sample metasurface QOM A where the polarization is kept along the LN optic axis. While at normal incidence in Fig. S3(a) we observe only two Mie-type resonances at 1591 nm and 1617 nm, with oblique incidence in Fig. S3(b,c) the situations drastically changes.

In case of excitation tilted in the xz-plane, Fig. S3(b), the electric resonance splits into two as clearly seen in Fig. S3(b)(ii) and becomes effectively wider. Additionally a new blue-shifted lattice resonance is appearing and moving towards shorter wavelengths with increasing angle of incidence.

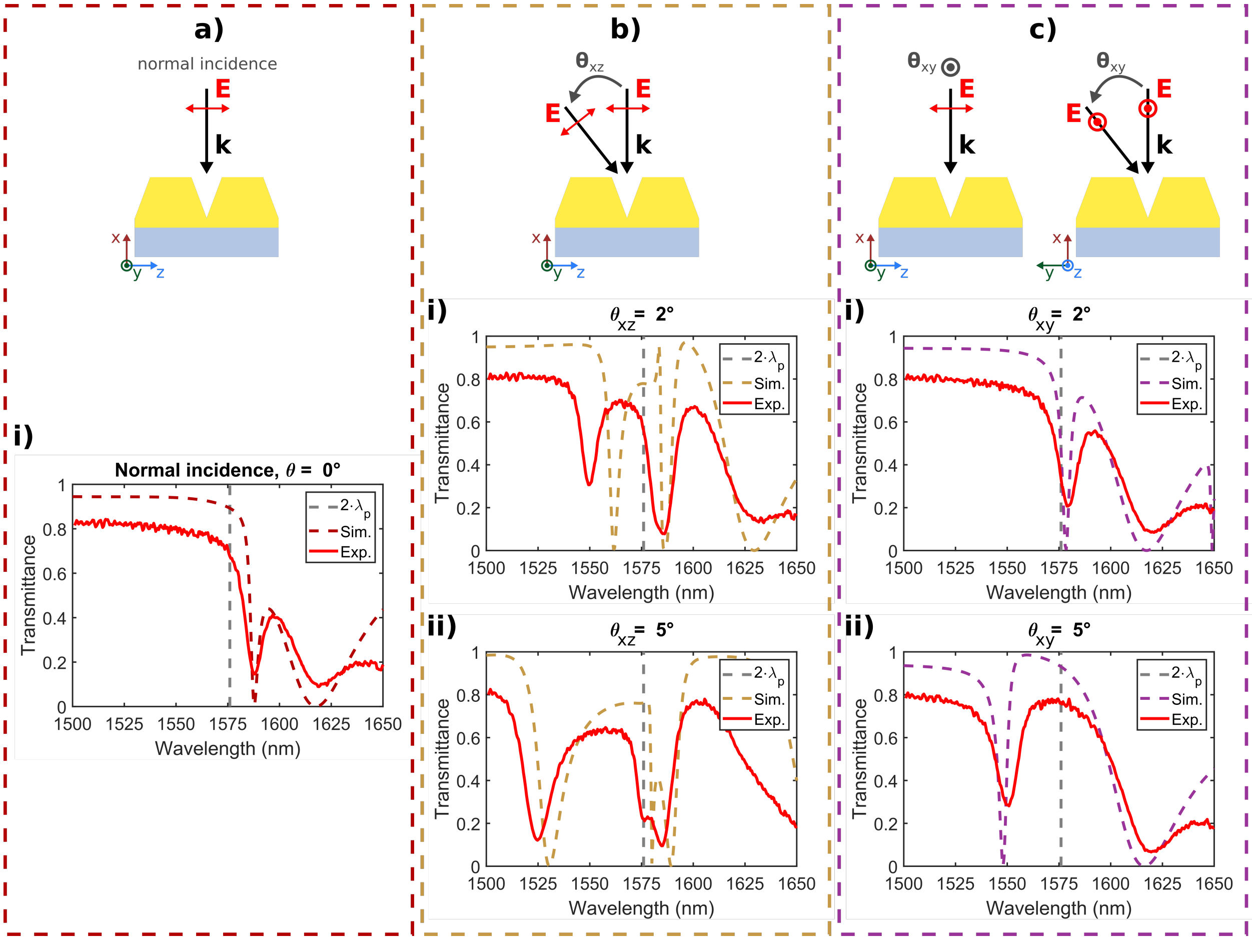

Figure S3: Experimental (solid lines) and simulated (dashed lines) transmittance spectra for QOM A, polarization along the LN optic axis. (a): normal incidence, (b): oblique incidence when excitation is tilted in the xz-plane (rotated around y-axis), (c): oblique incidence when the excitation is tilted in the xy-plane (rotated around z-axis); (i) and (ii): angle of incidence 2° and 5° respectively. The vertical dashed gray line indicates the degenerate photon-pair wavelength.

For the excitation tilted in the xy-plane, Fig. S3(c), the electric resonance moves to the shorter wavelengths by ≈10 nm for 2° incidence angle. In case of QOM A where its position at normal incidence 1591 nm is red-shifted in relation to the degenerate photon-pair wavelength $\lambda_{\mathrm{deg}} = 2 \cdot \lambda_{\mathrm{p}} = 1576$ nm, this means that the electric resonance moves closer to the degeneracy (at $\theta_{xy} = 2°$ it is at 1581 nm). This leads to more efficient sum-frequency generation (SFG) when signal and idler are incident under a small angle, as confirmed in the top panel of Fig. 4(b). Following the quantum-classical correspondence, SPDC for photon pairs emitted under a small angle should also be more efficient in QOM A.

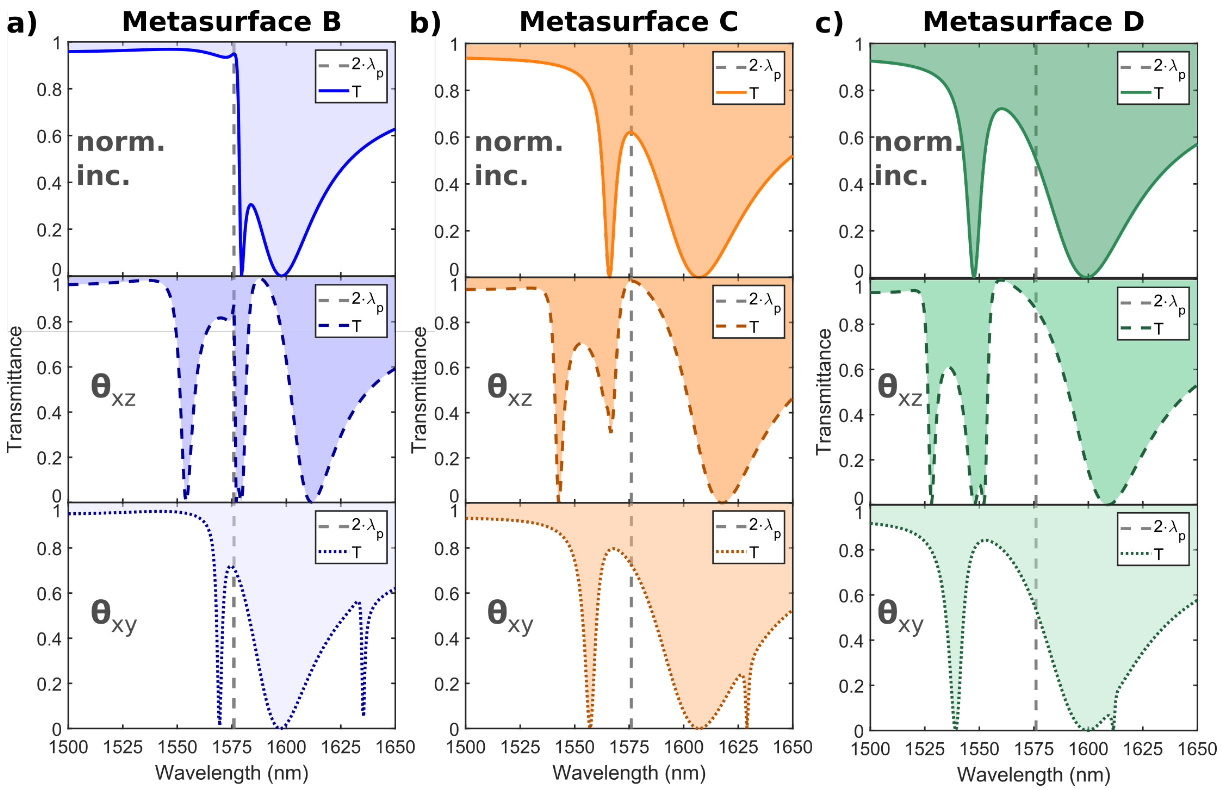

Figure S4: (a)-(c) Simulated transmittance spectra for QOMs B, C, D, respectively; for normal incidence (top row) and for the incidence angle 2° in the xz-plane (θ_{xz}, middle row) and in the xy-plane (θ_{xy}, bottom row). Polarization along the LN optic axis, z-axis. The vertical dashed gray line indicates the degenerate photon-pair wavelength.

For other QOMs the tendency in the linear spectra is the same (see Fig. S4): under oblique incidence the electric resonance shifts to shorter wavelengths. But since for QOM B the electric resonance is very close to λ_{deg} and for QOMs C and D the electric resonances are already blue-shifted with respect to λ_{deg}, upon increasing the angle of incidence they will move even further away from the degenerate wavelength. Thus, in these metasurfaces SFG/SPDC will only decrease at larger angles of incidence/emission.

Measurements with oblique incidence were performed by rotating the sample around its y-axis with a manual rotation stage (Fig. S3(b)) and around its z-axis with a motorized rotation stage (Fig. S3(c)). The manual stage was less precise which led to the discrepancy between the experimental curves and simulations in Fig. S3(b). But the tendency is still clear.

3 Multipole decomposition

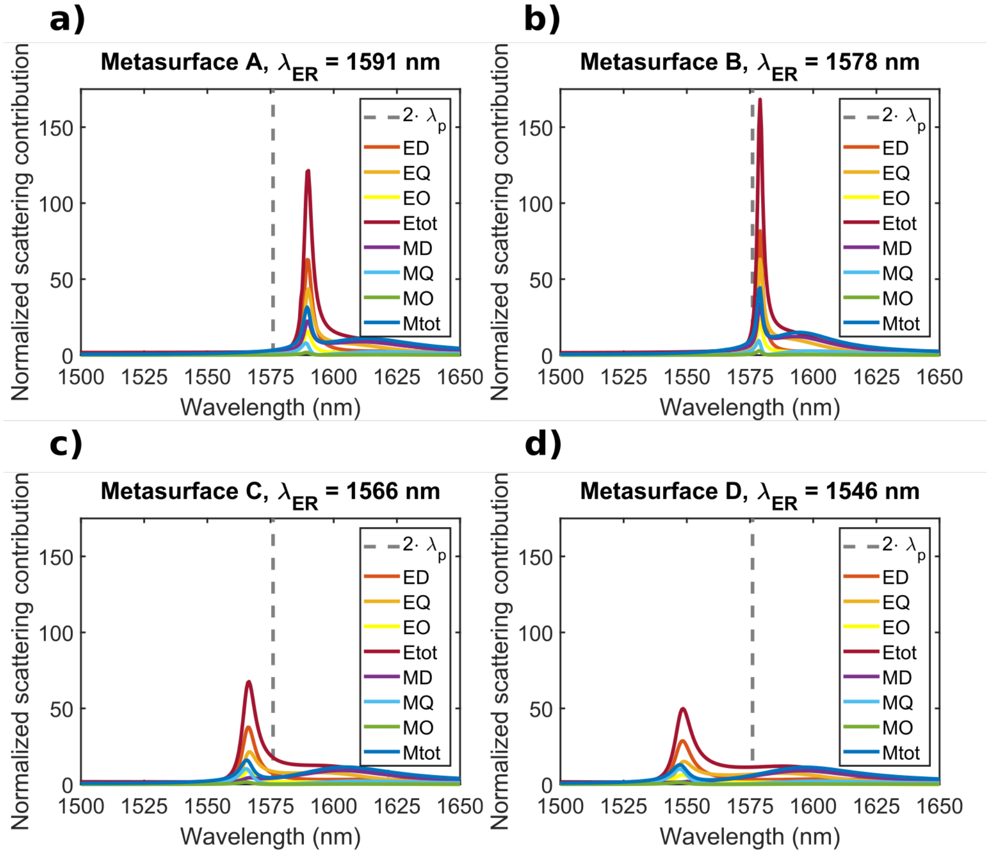

Figure S5: Multipole decomposition for QOMs of interest for polarization along the LN optic axis. The dashed gray line indicates the degenerate photon-pair wavelength. ED: electric dipole, EQ: electric quadrupole, EO: electric octupole, Etot: sum of ED, EQ, EO; MD: magnetic dipole, MQ: magnetic quadrupole, MO: magnetic octupole, Mtot: sum of MD, MQ, MO.

To perform multipole decomposition we used the method described in Ref. 2. As shown in Fig. S5, the resonances at shorter wavelengths are dominated by electric dipole and quadrupole contributions (therefore they are called electric), whereas the resonances at longer wavelengths are dominated by the magnetic dipole (therefore they are called magnetic). As discussed in the Supporting Information of Ref. 3, the multipole decomposition is very sensitive to the geometric parameters and can give only a general impression of the resonance's multipolar composition.

4 Field enhancement

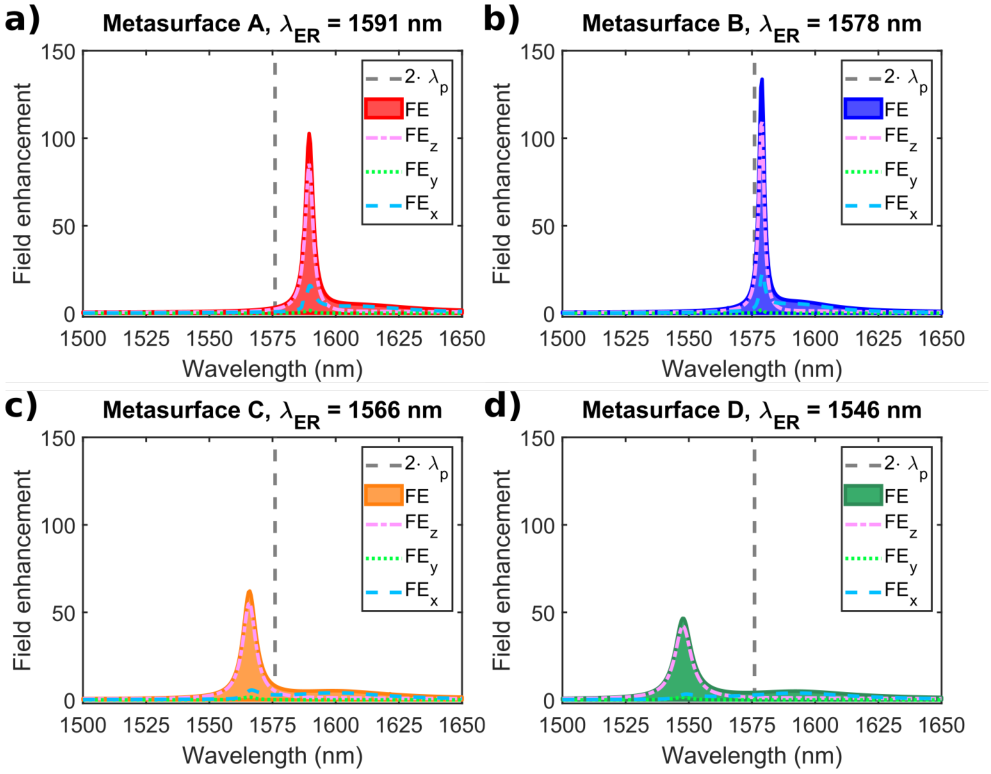

Figure S6: Field enhancement FE inside one pyramid for QOMs of interest, polarization along the LN optic axis, z-axis. The dashed gray line indicates the degenerate photon-pair wavelength. $\mathrm{FE_x}$, $\mathrm{FE_y}$, $\mathrm{FE_z}$ are field enhancements of x, y or z electric field component respectively.

Additionally, we calculated the field enhancement[4] inside our QOMs as

$$\mathrm{FE_i} = \frac{1}{V_\mathrm{res}} \int\limits_{V_\mathrm{res}} \frac{|E_i|^2}{E_0^2} dV, \qquad (1)$$

where V_res is the volume of one LN nanoresonator, $i = x, y, z$ and E_i is the corresponding electric field component, and E_0 is the amplitude of the incident electric field. In Fig. S6 we plot these components as well as the total field enhancement (solid lines with shading below them): $\mathrm{FE} = \mathrm{FE_x} + \mathrm{FE_y} + \mathrm{FE_z}$.

Clearly, at the electric resonances the z-component of electric field contributes the most to the total field enhancement; but at the magnetic resonances the x-component becomes

dominant. The field enhancement at the electric resonances is at least 10 times larger ($\approx$10 times for the QOMs C, D and $\approx$20-25 times for the QOMs A, B) than at the magnetic resonances. These simulations demonstrate that the highest field enhancement is at the electric resonance where the electric field component along the lithium niobate optic axis (z-component) is enhanced the most. This supports our experimental results: the electric resonances are more efficient for spontaneous parametric down-conversion (SPDC). Thus, in our QOMs we are able to fully utilize the lithium niobate's largest second-order susceptibility tensor element $\chi^{(2)}_{zzz}$.

5 Correlation experiment in reflection

We pumped the nonlinear metasurfaces with a cw pig-tailed diode laser delivering up to $\sim 70\,\mathrm{mW}$ at 788 nm. The pump power was controlled using a half-wave plate (HWP) and a polarizing beam-splitter (PBS). We used another HWP to rotate the pump polarization. The pump beam with a diameter of $\sim 2.5\,\mathrm{mm}$ was focused into the metasurfaces using a 90° off-axis gold-coated parabolic mirror with 15 mm reflective focal length. The same parabolic mirror collected the backward-generated SPDC radiation. The parabolic mirror played an essential role as, being free from chromatic aberrations, it ensured the collection of photon pairs from the same point where the pump laser was focused.

A longpass dichroic mirror with cut-on wavelength at 950 nm split the SPDC radiation and the portion of the pump laser reflected at the sample surface, with the SPDC being transmitted. Another longpass filter with cut-on wavelength at 1400 nm filtered out the remaining pump and removed most part of the fluorescence. We detected degenerate photon pairs after a 50 nm FWHM bandpass filter centered at 1575 nm. A broadband linear film polarizer oriented parallel to the LN optic axis selected photon pairs with z polarization. The polarizer also served as an additional filter removing fluorescence.

A parabolic mirror identical to the one used for focusing and collection fed the radiation

into the input facet of a 50:50 broadband single-mode fiber splitter (1550 nm ± 100 nm). Due to the identity of the two parabolic mirrors, the NA of the fiber (0.14) also determined the collection angle of SPDC. The two outputs of the fiber splitter were sent to infrared superconducting nanowire single-photon detectors (SNSPD). We registered the arrival time differences between the two detectors using a Swabian Instruments time-tagger (not shown).

6 Simulations of sum-frequency generation

Simulations of sum-frequency generation were done in COMSOL Multiphysics using the undepleted pump approximation, and included three steps. The first two are linear simulations of electromagnetic field for a plane wave excitation at (1) the signal frequency ω_s and (2) the corresponding idler frequency $\omega_i = \omega_p - \omega_s$, where ω_p is the frequency of our pump laser. Based on the electric fields from the first two steps, we calculated the nonlinear polarization (see Eq. 2) inside a LN nanoresonator (pyramid and residual layer, see Fig. S1) which in turn served as a source for the final SFG simulation. This algorithm was repeated by varying the signal frequency and setting the idler frequency accordingly. For the simulations of SFG with oblique incidence of signal and idler the excitation at the first step (signal study) was tilted by 2° and at the second step (idler study) by −2°, both either in xy or in xz planes (see sketches in Fig. S3). The components of the nonlinear polarization had the form[5]

$$\begin{aligned}
P_x^{NL}(\omega_p) &= 4\varepsilon_0 \left(d_{31}[E_x(\omega_s)E_z(\omega_i) + E_z(\omega_s)E_x(\omega_i)] - d_{22}[E_x(\omega_s)E_y(\omega_i) + E_y(\omega_s)E_x(\omega_i)]\right), \\
P_y^{NL}(\omega_p) &= 4\varepsilon_0 \left(d_{22}[E_y(\omega_s)E_y(\omega_i) - E_x(\omega_s)E_x(\omega_i)] + d_{31}[E_y(\omega_s)E_z(\omega_i) + E_z(\omega_s)E_y(\omega_i)]\right), \\
P_z^{NL}(\omega_p) &= 4\varepsilon_0 \left(d_{31}[E_x(\omega_s)E_x(\omega_i) + E_y(\omega_s)E_y(\omega_i)] + d_{33}E_z(\omega_s)E_z(\omega_i)\right), \qquad (2)
\end{aligned}$$

where $E_{x,y,z}(\omega)$ were the components of the signal (ω_s) or idler (ω_i) electric field, and $d_{22} = 1.9\,\mathrm{pm/V}, d_{31} = -3.2\,\mathrm{pm/V}, d_{33} = \frac{1}{2}\chi^{(2)}_{zzz} = -19.5\,\mathrm{pm/V}$ at 1313 nm.[6]

The resulting SFG spectra were multiplied by the SPDC spectrum from the wafer (gray

stars in Fig. 4(a)), to take into account the detectors sensitivity, and convolved with a Gaussian with FWHM 8.8 nm, to take into account the detectors timing jitter.

References

(1) Okoth, C.; Kovlakov, E.; Bönsel, F.; Cavanna, A.; Straupe, S.; Kulik, S. P.; Chekhova, M. V. Idealized Einstein-Podolsky-Rosen states from non–phase-matched parametric down-conversion. *Phys. Rev. A* **2020**, *101*, 011801.

(2) Grahn, P.; Shevchenko, A.; Kaivola, M. Electromagnetic multipole theory for optical nanomaterials. *New Journal of Physics* **2012**, *14*, 093033.

(3) Fedotova, A.; Younesi, M.; Sautter, J.; Vaskin, A.; Löchner, F. J.; Steinert, M.; Geiss, R.; Pertsch, T.; Staude, I.; Setzpfandt, F. Second-Harmonic Generation in Resonant Nonlinear Metasurfaces Based on Lithium Niobate. *Nano Letters* **2020**, *20*, 8608–8614, PMID: 33180501.

(4) Carletti, L.; Li, C.; Sautter, J.; Staude, I.; De Angelis, C.; Li, T.; Neshev, D. N. Second harmonic generation in monolithic lithium niobate metasurfaces. *Optics Express* **2019**, *27*, 33391–33398.

(5) Boyd, R. W. *Nonlinear Optics*; Elsevier: New York, 2008.

(6) Shoji, I.; Kondo, T.; Kitamoto, A.; Shirane, M.; Ito, R. Absolute scale of second-order nonlinear-optical coefficients. *JOSA B* **1997**, *14*, 2268–2294.
